# Aberrant Cellular Glycosylation May Increase the Ability of Influenza Viruses to Escape Host Immune Responses through Modification of the Viral Glycome

**DOI:** 10.1128/mbio.02983-21

**Published:** 2022-03-14

**Authors:** Irina V. Alymova, John F. Cipollo, Lisa M. Parsons, Nedzad Music, Ram P. Kamal, Wen-Pin Tzeng, Cynthia S. Goldsmith, Joseph N. Contessa, Kevan L. Hartshorn, Jason R. Wilson, Hui Zeng, Shane Gansebom, Ian A. York

**Affiliations:** a Immunology and Pathogenesis Branch, Influenza Division, National Center for Immunization & Respiratory Diseases, Centers for Disease Control & Prevention, Atlanta, Georgia, USA; b Food and Drug Administration, Center for Biologics Evaluation and Research, DBPAP, Silver Spring, Maryland, USA; c Battelle Memorial Institute, Atlanta, Georgia, USA; d Infectious Diseases Pathology Branch, Division of High-Consequence Pathogens and Pathology, National Center for Emerging and Zoonotic Infectious Diseases, Centers for Disease Control & Prevention, Atlanta, Georgia, USA; e Department of Therapeutic Radiology, Yale School of Medicine, New Haven, Connecticut, USA; f Department of Pharmacology, Yale School of Medicine, New Haven, Connecticut, USA; g Department of Medicine, Boston University School of Medicine, Boston, Massachusetts, USA; h Carter Consulting, Inc., Atlanta, Georgia, USA; The Peter Doherty Institute for Infection and Immunity

**Keywords:** virus, influenza, hemagglutinin, neuraminidase, pathogenicity, NGI-1, glycosylation, immunity

## Abstract

Individuals with metabolic dysregulation of cellular glycosylation often experience severe influenza disease, with a poor immune response to the virus and low vaccine efficacy. Here, we investigate the consequences of aberrant cellular glycosylation for the glycome and the biology of influenza virus. We transiently induced aberrant N-linked glycosylation in cultured cells with an oligosaccharyltransferase inhibitor, NGI-1. Cells treated with NGI-1 produced morphologically unaltered viable influenza virus with sequence-neutral glycosylation changes (primarily reduced site occupancy) in the hemagglutinin and neuraminidase proteins. Hemagglutinin with reduced glycan occupancy required a higher concentration of surfactant protein D (an important innate immunity respiratory tract collectin) for inhibition compared to that with normal glycan occupancy. Immunization of mice with NGI-1-treated virus significantly reduced antihemagglutinin and antineuraminidase titers of total serum antibody and reduced hemagglutinin protective antibody responses. Our data suggest that aberrant cellular glycosylation may increase the risk of severe influenza as a result of the increased ability of glycome-modified influenza viruses to evade the immune response.

## INTRODUCTION

Glycosylation of newly synthesized proteins is an important part of cellular function in eukaryotic organisms. Individuals with a variety of conditions, including cancer, autoimmune disease, diabetes, or obesity may have metabolic dysregulation of cellular glycosylation, so that some proteins are not properly glycosylated (1–6). These conditions are also associated with reduced immune responses to influenza virus, more severe disease following influenza infection, and faster waning of protection from the influenza vaccine (7–14). A number of host factors have been linked to the blunted immune response to influenza virus infection under these conditions, including systemic alterations in both the innate and adaptive immune systems (9, 10, 15–17). In contrast, relatively little is known about changes in the infecting virus.

Influenza virus utilizes host cell asparagine (N)-linked glycosylation (here, referred to as “N-glycosylation”) to glycosylate its surface proteins, hemagglutinin (HA), and neuraminidase (NA). Proper N-glycosylation is critical for the biological functions of HA and NA (such as receptor binding and sialidase enzymatic activity, respectively) and for triggering both innate and adaptive immune responses to the virus in the host (18–23). Genomic sequences of influenza viruses isolated from people with imbalanced metabolic glycosylation generally do not show consistent or specific variations, including those in the N-linked sequon Asn-(X≠Pro)-Ser/Thr (24, 25). However, it remains possible that viruses infecting these people may undergo abnormal glycosylation (as with host proteins), without genome sequence changes, and that these glycomic changes can make the virus less vulnerable to host immune responses.

N-glycosylation in mammalian cells is catalyzed by the oligosaccharyltrasferase (OST) enzyme complex in the endoplasmic reticulum, followed by trimming and remodeling of the oligosaccharides during transit through the Golgi apparatus. In the case of influenza virus HA and NA, the final products of the process are glycoproteins whose N-linked oligosaccharide branched structures terminate in mannose (high-mannose glycans), galactose and/or *N*-acetylglycosamine/fucose (complex glycans), or a combination of these (hybrid glycans) (26, 27).

Mammalian OST is a heterooligomer composed of a single copy of a catalytic subunit (either STT3A or STT3B) and a shared set of six noncatalytic subunits and complex-specific accessory subunits (28–31). The STT3A subunit transfers oligosaccharides onto asparagine residues and provides the majority of N-glycosylation in mammalian cells, while STT3B glycosylates only a small number of sequons but increases site occupancy of sites that have been missed by STT3A (32). Genetic deficiencies of either the STT3A or STT3B expression cause severe type I congenital disorders of glycosylation of a similar severity (33). Increased or reduced expression of either OST subunit can be present in cancers, including those of the human respiratory tract (34–37).

Treatment with a small-molecule inhibitor of mammalian OST, NGI-1, significantly reduced N-hyperglycosylation of proteins in some lung cancer cells (36). NGI-1 reversibly and without generalized toxicity blocks STT3B-dependent, and partially inhibits STT3A-dependent, glycosylation and is generally nontoxic to cell cultures at concentrations that effectively inhibit OST function (36–38). NGI-1 was previously used to study the life cycle of flaviviruses (39), herpes simplex virus 1 (40), and Lassa virus (41) under conditions of transient N-hypoglycosylation.

Here, we treated cultured cells with NGI-1 to produce viable influenza virus with glycosylation changes in HA and NA to determine the effect of these glycosylation changes on influenza virus biology and on virus interactions with the innate and adaptive immune systems.

## RESULTS

### Treatment of primary and cultured cells with NGI-1 reduces growth of influenza A and B viruses.

**FIG 1:**
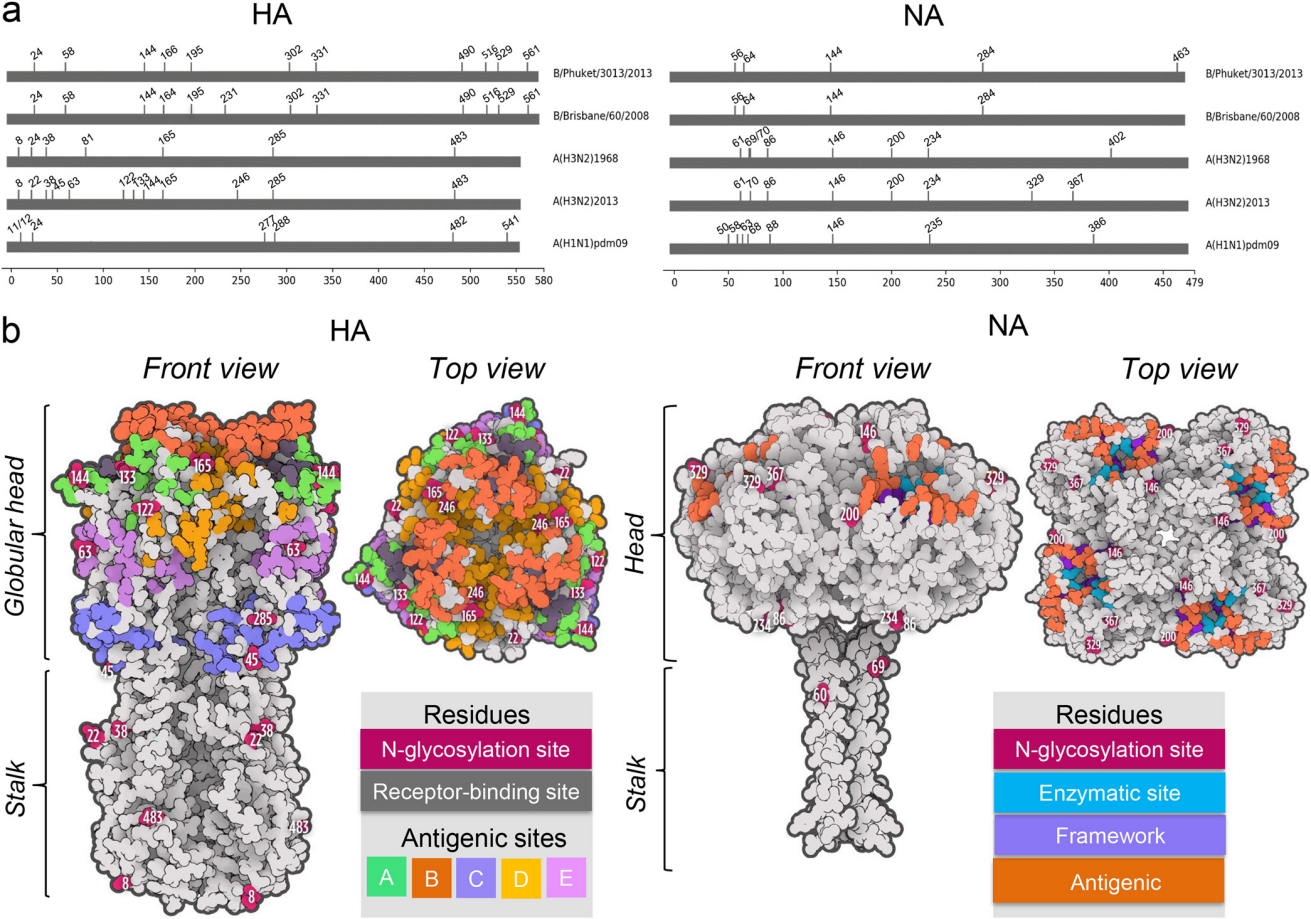
N-glycosylation sites of influenza viruses. (a) Sites of putative N-glycosylation of the hemagglutinin (HA) and the neuraminidase (NA) were identified based on sequon Asn-(X≠Pro)-Ser/Thr. The numbers indicate the start of an N-glycosylation sequon (Asn). Numbering of HA N-glycosylation sites is based on the mature molecule with the 16-amino acid signal sequence cleaved. (b) Structure, N-glycosylation, and antigenic sites of the A(H3N2)2013 IAV HA and NA. Shown are the surface area representations of HA and NA (based on PDB accession numbers 6PDX and 3TIC, respectively) (81, 82).

We examined the effect of OST inhibition with NGI-1 on the growth of human influenza A (H3N2 and H1N1 subtypes) and B viruses (IAV and IBV, respectively) (Fig. 1) in primary normal human bronchial epithelial (NHBE) cells (which maintain human airway characteristics) and Madin-Darby canine kidney (MDCK) epithelial cells (commonly used for infection with influenza virus). Influenza A(H1N1)pdm2009 and A(H3N2)1968 each have 7 putative N-glycosylation sites on HA, while A(H3N2)2013 and B/Brisbane/60/2008 (Victoria lineage) have 12, and B/Phuket/3073/2013 (Yamagata lineage) has 11 (Fig. 1a and b). There are 8 N-glycosylation sites in the NA of each of the IAV, while that of B/Brisbane/60/2008 contains 4, and of B/Phuket/3073/2013 has 5 (Fig. 1a and b).

Cells were treated with 10.0 μM NGI-1 once, either 1 h prior to infection or up to 24 h postinfection (p.i.), with the NGI-1 remaining in the cell culture supernatant until the end of the experiment. This NGI-1 concentration did not reduce the viability of either NHBE or MDCK cells based on trypan blue exclusion assays (Fig. S1), consistent with observations of other cell lines (36, 37).

10.1128/mBio.02983-21.1 FIG S1 NGI-1 exhibits low cytotoxicity in NHBE and MDCK cell monolayers. (a) NHBE or (b) MDCK cell monolayers were treated with 10.0 μM NGI-1 for 24, 48, or 72 h, and the percentage of viable cells was determined compared to the total number of cells for each sample. Values are shown as the mean ± SD of the results from at least three cultures. Differences between NGI-1-treated and untreated samples were not significant (*P* > 0.05). Download FIG S1, TIF file, 1.0 MB. Copyright © 2022 Alymova et al. 2022 Alymova et al. https://creativecommons.org/licenses/by/4.0/ This content is distributed under the terms of the Creative Commons Attribution 4.0 International license.

**FIG 2:**
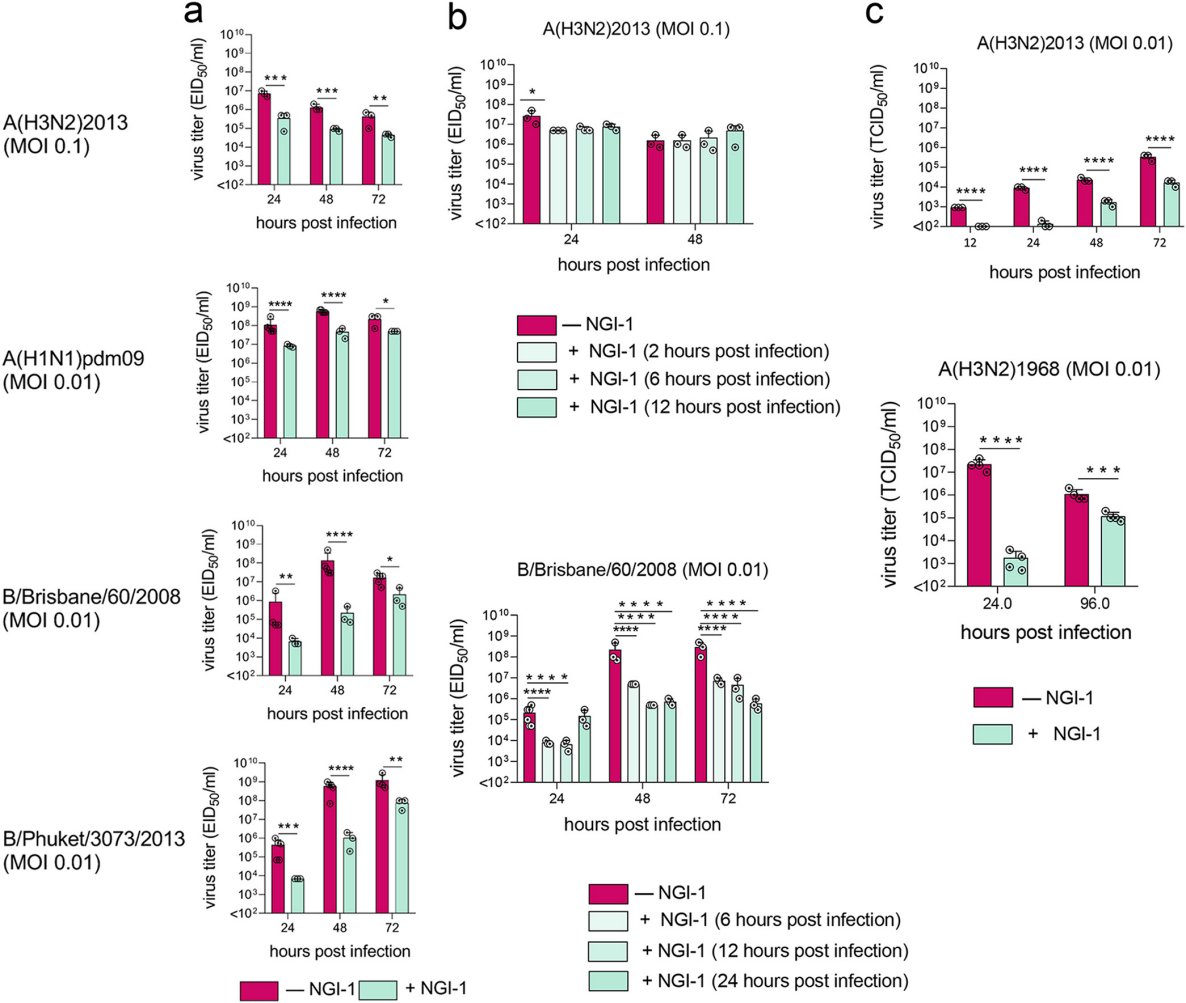
OST inhibition reduces infectious titers of IAV and IBV in epithelial cells. (a to c) NGI-1 treatment (10.0 μM) of (a and b) NHBE or (c) MDCK cell monolayers was initiated either (a and c) 1 h prior or (b) 2 h to 24 h postinfection. Viral titers of apical culture supernatant fluids were assessed at the indicated time points. Error bars indicate the standard deviation (SD) of the mean of the results from at least (a and b) three cultures or (c) three biological replicates in one representative experiment (of three total). Significant differences: *, *P* < 0.05; **, *P* < 0.005; ***, *P* < 0.0005; ****, *P* < 0.00005. MOI, multiplicity of infection.

Treatment of NHBE cells with NGI-1 led to significantly reduced the 50% egg or tissue culture infectious dose per mL (EID_50_/mL and TCID_50_/mL, respectively) titers of all tested influenza viruses whether the inhibitor was added before or after infection (Fig. 2a and b). Infectious titers of both IBV viruses in the culture supernatants were reduced up to 1,000-fold compared to those of untreated cells by the pretreatment, while titers of both IAV in the culture supernatant were reduced up to 10-fold (Fig. 2a). IBV titers were also reduced more than those of IAV when NGI-1 was added to cells up to 24 h p.i. (Fig. 2b). As with NHBE cells, pretreatment of MDCK cells with NGI-1 reduced infectious titers of both IAV (Fig. 2c).

**FIG 3:**
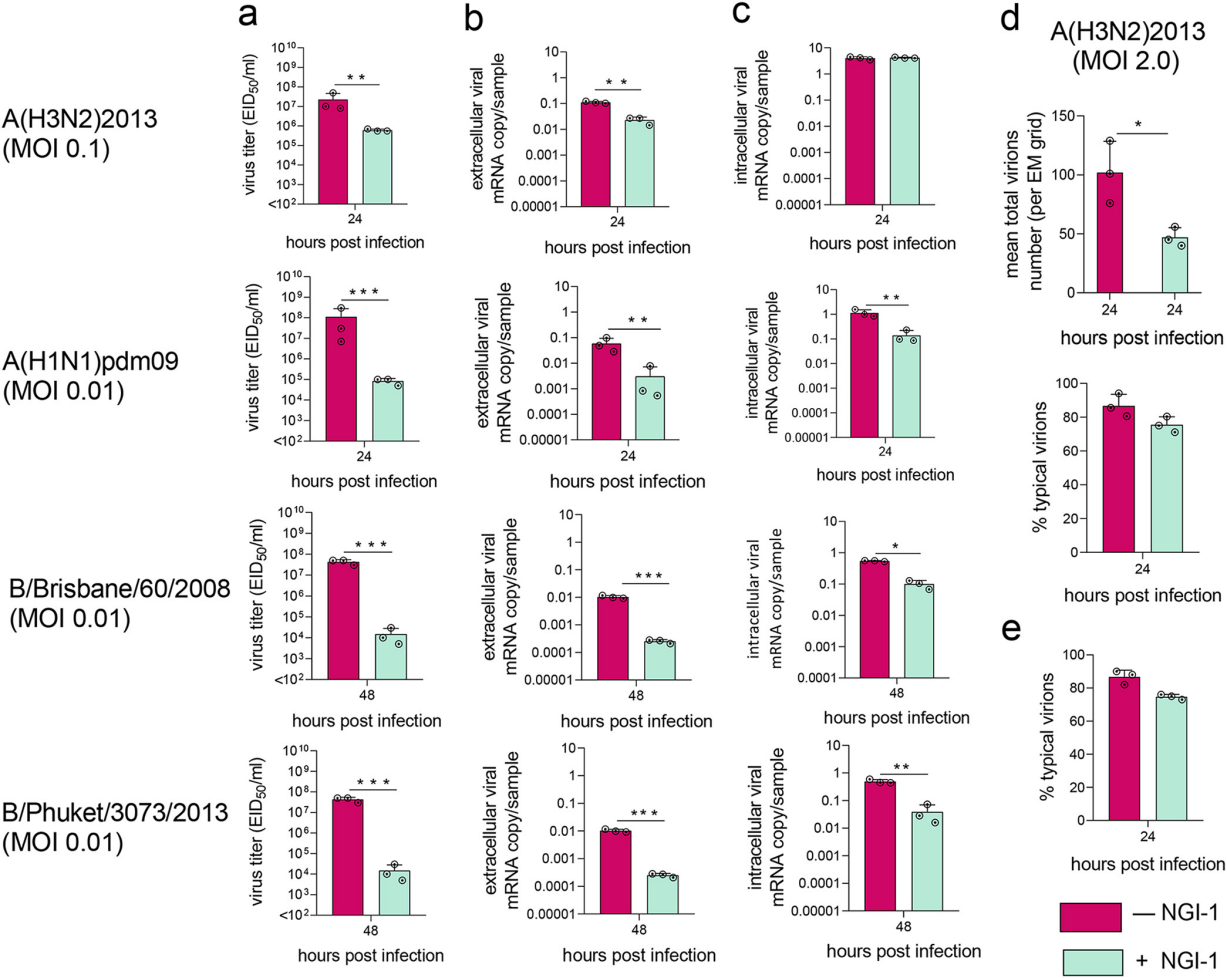
OST inhibition reduces RNA levels and total virion number but does not affect the morphology of IV in epithelial cells. (a to d) NHBE or (e) MDCK cell monolayers were pretreated with (a to d) 10.0 μM or (e) 5.0 μM NGI-1 for 1 h prior to infection with IAV and IBV. (a) Viral infectious titers, (b) extracellular and (c) intracellular RNA levels, (d) number of total and (d and e) morphologically typical virions (counted by electron microscopy) in (a, b, d, and e) apical culture supernatant fluids were examined at the indicated times. Values are shown as the mean ± SD of the results from at least three cultures. Significant differences: *, *P* < 0.05; **, *P* < 0.005, ***, *P* < 0.0005. MOI, multiplicity of infection.

N-glycosylation of influenza virus is required for proper viral binding and release (42, 43). Reverse transcription quantitative PCR (RT-qPCR) was used to determine the levels of intra- and extracellular viral RNA from NHBE cells pretreated with 10.0 μM NGI-1. As with previous experiments, infectious titers of extracellular IAV and IBV were reduced upon NGI-1 treatment (Fig. 3a). Consistent with this, extracellular viral RNA levels were also decreased following NGI-1 treatment (Fig. 3b). Intracellular RNA was also reduced, though to a lesser extent, for three of the four tested viruses; intracellular RNA levels of A(H3N2)2013 were not significantly affected (Fig. 3c).

The impact of this alteration on viable A(H1N1)pdm09 and IBV was at least 10-fold greater than on virus genome production (compare Fig. 3a and b), suggesting that not only were fewer virions produced in the presence of NGI-1, but that the average infectivity of these viral particles was reduced. In contrast, infectivity of A(H3N2)2013 was not affected by NGI-1 treatment, since infectious and extracellular genomic viral titers were reduced to a similar extent (about 10-fold).

We performed more detailed studies using H3N2 IAV, the most prevalent subtype during influenza season, with severe impacts on human morbidity and mortality (44). An electron microscope was used to count A(H3N2)2013 virions in culture supernatant. NHBE cells pretreated with 10.0 μM NGI-1 had approximately 2.5 times fewer total virions than supernatant from untreated cells (Fig. 3d), consistent with the reduced infectious and extracellular viral genomic titers (Fig. 3a and b). However, both samples contained typical spherical and filamentous viral particles, at similar frequencies (Fig. 3d). Similarly, treatment of MDCK cells with NGI-1 did not affect A(H3N2)2013 virions’ morphology (Fig. 3e).

A(H3N2)2013 IAV grown in NHBE cells with NGI-1 was functional, since infection of untreated MDCK cells at a multiplicity of infection (MOI) of 0.01, or of eggs with 4 hemagglutination units (HAU), with NGI-1-treated or -untreated virus led to similar infectious viral titers at 24 h p.i. to 72 h p.i. (for cell infections) (Fig. S2a) or 48 h p.i. (for egg infections) (Fig. S2b). Similarly, A(H3N2)2013 IAV grown in NGI-1-treated MDCK cells showed efficient growth in untreated MDCK cells (Fig. S2c).

10.1128/mBio.02983-21.2 FIG S2 H3N2 IAV from NGI-1-treated cells exhibits normal growth at conditions of unmodified N-glycosylation. NHBE or MDCK cell monolayers were untreated or pretreated with 10.0 μM NGI-1 1 h prior to infection with A(H3N2)2013 IAV. Virus-containing apical culture supernatants were collected 24 h postinfection, and viral titers were determined. (a) MDCK cell monolayers or (b) 10-day-old embryonated chicken eggs with unmodified N-glycosylation were infected with A(H3N2)2013 IAV from untreated or NGI-treated NHBE cells at MOI 0.01 (in the case of MDCK cells) or with 4 HA (in the case of embryonated chicken eggs). (c) MDCK cells with normal N-glycosylation were infected with untreated or NGI-treated MDCK cells grown with virus at MOI 0.01. Viral titers were assessed at the indicated time points. Data represent the average (± SD) of the results from one representative experiment (of two total). Differences between NGI-1-treated and untreated samples were not significant (*P* > 0.05). Download FIG S2, TIF file, 0.8 MB. Copyright © 2022 Alymova et al. 2022 Alymova et al. https://creativecommons.org/licenses/by/4.0/ This content is distributed under the terms of the Creative Commons Attribution 4.0 International license.

These data demonstrate that inhibition of OST with NGI-1 can reduce infectious and genomic IAV and IBV titers without changing virion morphology or the virus’s ability to infect normal cells.

### OST inhibition produces H3N2 IAV with reduced glycosylation.

**FIG 4:**
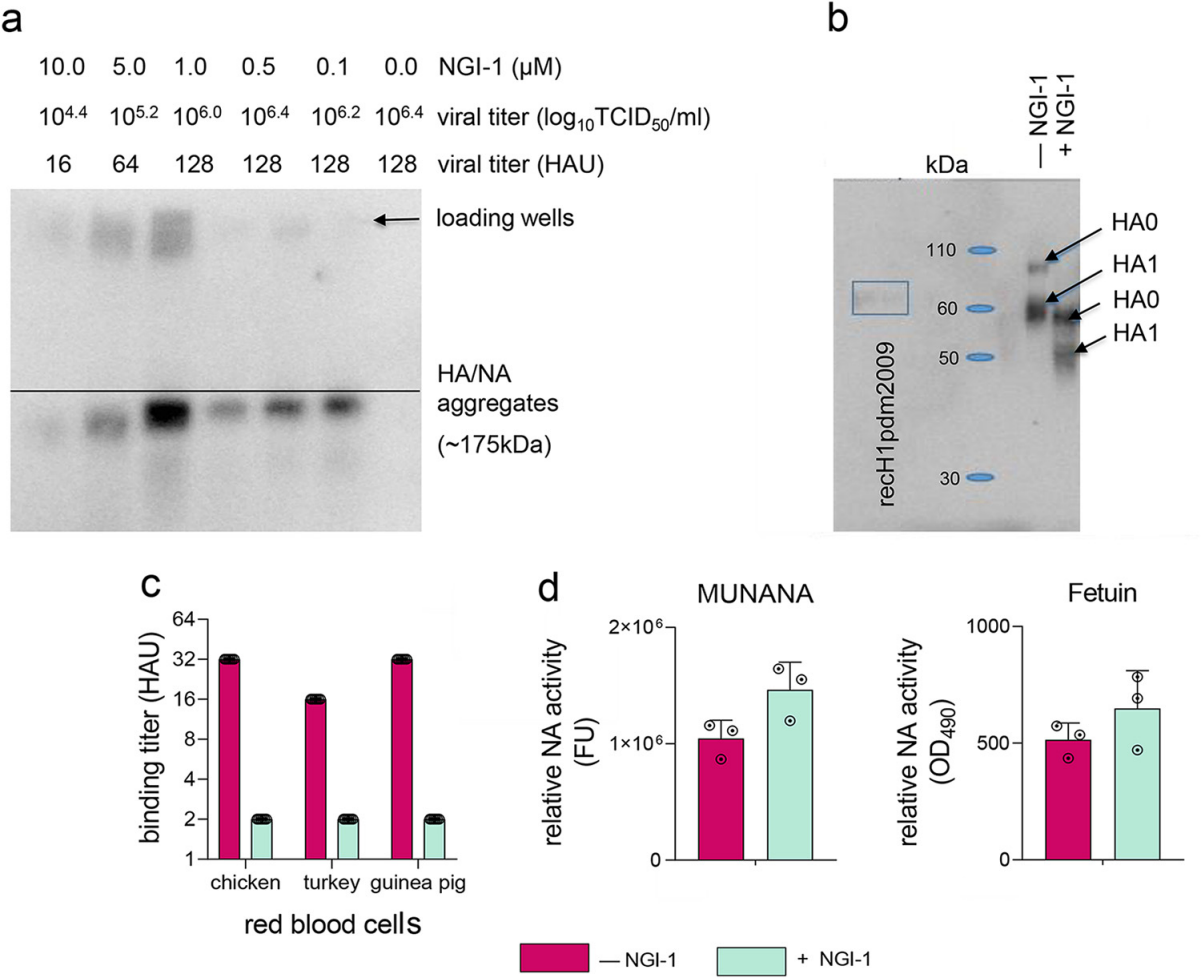
Electrophoretic mobility, receptor binding, and enzymatic activity of NGI-1-treated H3N2 IAV proteins. (a to d) MDCK cells were untreated or pretreated with NGI-1 at (a) the indicated dosages, (b and c) 5.0 μM, or (d) 10.0 μM 1 h before infection with A(H3N2)2013 IAV at (a to c) MOI 2.0 or (d) MOI 0.1. (a) Viral titers from culture supernatants and migration patterns of the HA/NA aggregates from cell lysates were determined 24 h postinfection. (b) Immunoblot analysis of HA from concentrated purified A(H3N2)2013 IAV collected 24 h postinfection from untreated or NGI-1-treated MDCK cells. Molecular mass standards in kilodaltons (kDa) are indicated. Secreted recombinant H1pdm2009 protein, whose predicted molecular mass is similar of that of secreted recombinant H3N2 HA (60 kDa and 59.1 kDa, respectively) as a control was provided by J. Stevens (CDC, Atlanta, GA, USA). (c) Binding to red blood cells of glycome-modified or unmodified concentrated purified A(H3N2)2013 IAV normalized to HA mass. The hemagglutination unit (HAU) titers were determined after incubation of virus with red blood cells for 1 h at 4°C. The means of two independent experiments performed in triplicate are shown. (d) Enzymatic activity of NA from glycome-modified or unmodified A(H3N2)2013 IAV collected 24 h postinfection from culture supernatant was determined by using MUNANA or fetuin as a substrate, normalized to the viral mRNA copy number. One representative experiment of two independent experiments, performed in triplicate, is shown. The mean enzymatic activity of NA ± SD is shown.

A(H3N2)2013 IAV was propagated in MDCK cells (MOI, 2.0) that were pretreated for 1 h with NGI-1 at concentrations between 0.1 and 10.0 μM. After 24 h, infectious virus titers and electrophoretic mobility of HA and NA from lysates of virus-infected treated versus untreated cells were compared. Titers of the A(H3N2)2013 IAV in culture supernatant (measured by both TCID_50_/mL and HAU/mL) were progressively reduced with increasing NGI-1 doses, while the gel electrophoretic mobility of NGI-1-treated HA and NA was increased (Fig. 4a and b). Genome sequencing showed no mutations in the HA and NA of the virus isolated from NGI-1-treated cells compared to virus from untreated cells, and sequences of viruses from both treated and untreated cells matched the expected sequences (Global Initiative on Sharing Avian Influenza Data [GISAID] accession number EPI_ISL_207641). The changes in viral titers and proteins’ electrophoretic mobility were therefore presumably due to a loss of glycans on the proteins rather than sequence variants.

Mass spectrometry was used to analyze the extent and nature of N-glycosylation on HA and NA from concentrated purified A(H3N2)2013 IAV that had been grown in MDCK cells pretreated with 5.0 μM NGI-1 or untreated. All 12 HA N-glycosylation sites (Fig. 1b) of A(H3N2)2013 IAV from untreated and NGI-1-treated cells contained glycans (Table 1). Half of them showed a significant reduction in glycosylation when virus was grown with NGI-1. Although NetNGlc predicted glycan occupancies at NA sites N61 and N70 (potentials of 0.7567 and 0.5453), no significant intact glycopeptide ion intensities were observed for these sites. However, site occupancy analyses did reveal that both sites are occupied, albeit with site N70 at reduced levels especially when expressed under NGI-1 inhibition. Most of the HA N-glycosylation sites with reduced occupancy were located within the globular head (N45, N63, N122, N165, and N246; numbering is based on the mature protein), while one (N483) was in the HA stalk region (Fig. 1b).

**TABLE 1:** N-glycosylation of NGI-1-treated H3N2 IAV hemagglutinin (HA) and neuraminidase (NA)^*a*^

HA or NA site	Untreated virus		NGI-1-treated virus		NetNGlyc potential probability score/hydrophobicity
	Glycoform(s)	Site occupancy (% ± SD)	Glycoform(s)	Site occupancy (% ± SD)	
HA site					
8(NST)	ND	100 ± 0.0	*dHex1Hex5HexNAc4* *dHex1Hex6HexNAc4* *dHex1Hex8HexNAc6*	87.7 ± 17.6	0.8062/30.42
22(NGT)	ND	100 ± 0.0	*dHex1Hex5HexNAc4* *dHex1Hex8HexNAc6*	87.7 ± 17.6	0.6976/30.42
38(NAT)	*Hex5HexNAc2*	100 ± 0.0	*Hex5HexNAc2* *dHex1Hex5HexNAc4* *Hex5HexNAc4*	63.6 ± 54.5	0.5357/50.17
45(NSS)	ND	32.6 ± 3.7	ND	8.1 ± 1.1***	0.5549/50.17
63(NCT)	ND	75.0 ± 21.7	ND	20.1 ± 0.7*	0.7091/50.17
122(NES)	*Hex7HexNAc2*	100 ± 0.0	*Hex4HexNAc2* *Hex7HexNAc2*	52.1 ± 0.1***	0.4407/35.98
133(NGT)	*Hex5HexNAc2* *Hex6HexNAc2* *Hex7HexNAc2*	100 ± 0.0	*Hex5HexNAc2* *Hex6HexNAc2* *Hex7HexNAc2*	ND	0.6482/35.98
144(NSS)	*dHex2Hex5* *HexNAc6* *dHex1Hex5* *HexNAc4*	22.5 ± 5.6	*dHex2Hex5HexNAc6* *dHex1Hex5HexNAc4* *Hex5HexNAc2*	24.5 ± 0.1	0.5288/18.5
165(NVT)	*Hex7HexNAc2* *Hex8HexNAc2* *Hex9HexNAc2*	21.3 ± 4.0	*Hex7HexNAc2* *Hex8HexNAc2*	11.3 ± 1.1*	0.8138/31.0
246(NST)	*Hex8HexNAc2* *Hex9HexNAc2*	100 ± 0.0	*Hex8HexNAc2*	52.6 ± 9.0**	0.5879/55.13
285(NGS)	*Hex7HexNAc2* *Hex8HexNAc2* *Hex9HexNAc2*	69.7 ± 3.0	*Hex6HexNAc2* *Hex7HexNAc2* *Hex8HexNAc2*	68.9 ± 13.1	0.6762/27.04
483(NGT)	ND	95.3 ± 2.7	*dHex1Hex6HexNAc5* *dHex1Hex7HexNAc5* *dHex1Hex5HexNAc4* *dHex1Hex6HexNAc4* *dHex1Hex4HexNAc3* *NeuAc2dHex1Hex4HexNAc4* *NeuAc2dHex1Hex6HexNAc3* *NeuAc1dHex1Hex7HexNAc3*	61.7 ± 1.5***	0.5203/12.89
NA site					
61(NIT/NTT)	ND	86.7 ± 4.5	ND	100 ± 0.0	0.7567/33.99
70(NTT)	ND	12.9 ± 2.4	ND	7.9 ± 2.9	0.5453/33.99
86(NWS)	*H8N2*	78.6 ± 4.0	*Hex7HexNAc2* *Hex8HexNAc2*	5.5 ± 1.7***	0.6021/33.72
146(NNT)	ND	100 ± 0.0	*dHex1Hex6HexNAc4* *dHex1Hex6HexNAc5* *dHex1Hex7HexNAc4* *dHex1Hex7HexNAc5* *dHex1Hex8HexNAc5*	45.2 ± 5.6***	0.5968/31.12
200(NAT)	ND	72.9 ± 6.1	ND	44.4 ± 14.1*	0.3680/22.8
234(NGT)	ND	ND	*dHex1Hex2HexNAc3*	ND	0.7558/25.19
329(NDS)	ND	ND	ND	ND	0.4883/9.75
367(NET)	ND	81.2 ± 2.6	ND	59.23 ± 3.0**	0.5408/5.18

a The N-glycosylation site occupancy and glycoform compositions of HA and NA of concentrated purified A(H3N2)2013 IAV grown in the presence or absence 5.0 μM NGI-1 were analyzed using LC/MS^E^-spectrometry analysis as described elsewhere (76). Numbers indicate the start of an N-glycosylation sequon (Asn) (83). In the case of HA, the numbering is based on mature molecules (without of the 16-amino acid signal). NA on influenza virions is present at approximately one-third the abundance of HA, and its lower abundance may have limited the detection of some glycoforms. The N-glycosylation potential is shown as the probability score and predicted by NetNGlyc 1.0 (78). The hydrophophobicity is derived from the Peptide Synthesis and Proteotypic Peptide Analyzing Tool. Identical hydrophobicity in neighboring N-glycosylation sites indicates that sites are on the same peptide. Data represent the average (± SD of the mean) of assays run in triplicates. Asterisks indicate significant differences in site occupancy between untreated and NGI-1-treated virus by Student’s unpaired *t* test. *, *P* < 0.05; **, *P* < 0.005; ***, *P* < 0.0005. ND, not determined.

Glycoforms detected at each site of HA were similar for A(H3N2)2013 IAV from NGI-1-treated or untreated cells (Table 1). N-glycosylation sites of HA containing primarily high-mannose glycans included N122, N133, N165, N246, and N285. Those containing compositions consistent with hybrid and complex glycans included glycosites N8, N22, N38, N144, and N483.

Glycosylation was detected at 7 of 8 predicted N-glycosylation sites for A(H3N2)2013 IAV NA based on the combined intact glycopeptide and site occupancy data (Table 1). Only low-ion-abundance glycopeptides were detected at glycosite N234 of NA. There was no ion abundance intensity associated with site N329 occupancy or glyopeptide. This may be partially due to the predicted low glycosylation potential of that site, combined with poor ionization; the tryptic peptide (NDSSSSSHCLDPNNEEGGHGVK) for this site is highly hydrophilic, which would predict poor ionization.

A reduction of N-glycosylation site occupancy was detected in 4 NA sites in virus grown in NGI-1-treated cells. Three sites with reduced occupancy (N146, N200, and N367) were in the NA head, and one (N86) was in the stalk region (Fig. 1b). Although the number of glycoforms detected in NA were limited, some trends can be observed. Glycosite N86 appears to contain primarily high-mannose glycans, and sites N146 and N234 contain primarily complex glycans (Table 1). Where glycoforms were detected in NA, they were of similar composition and subclass whether the virus was from cells treated with NGI-1 or not.

These data demonstrate that inhibition of normal catalytic activity of cellular OST reduces N-glycosylation site occupancy of influenza virus HA and NA, with no change in glycoforms.

### Glycome modifications reduce HA receptor binding but do not affect the enzymatic activity of NA.

The functions of influenza virus HA and NA involve interaction with sialic acid. The receptor-binding site of the HA globular head initiates infection by binding to specific α2,3- or α2,6-linked sialic acid-containing receptors on epithelial cells and mucins of the human respiratory tract (45). The catalytic site of the NA head cleaves sialic acid residues from viral and cellular glycoproteins and from mucins, thus preventing self-aggregation of newly formed virions at the surface of infected cells and enabling virus release from cells and movement within extracellular mucins to target cells (46–48).

N-glycosylation was reduced at sites N165 and N246 of HA of A(H3N2)2013 IAV isolated from NGI-1-treated cells (Table 1). These sites are close to residues 153, 155, 226, and 228, which along with other residues form the receptor-binding site (Fig. 1b) (49). In the case of NA, three of the four sites with reduced occupancy (N146, N200, and N367) were in the proximity of 118, 151, 152, 224, and 371 residues (which are among the key residues for the catalytic function) and 119, 156, 198, and 222 framework residues (which help to stabilize the structure of the NA catalytic site) (50, 51).

To test receptor binding of A(H3N2)2013 IAV with modified N-glycosylation, we prepared concentrated purified virus grown with or without NGI-1, normalized amounts of virus by HA mass, and performed hemagglutination assays using red blood cells (RBC) from chickens (abundant in α2,3-linked sialic acids) or turkeys or guinea pigs (abundant in α2,6-linked sialic acids) (52). In each case, the virus with unmodified N-glycosylation had higher binding titers per μg than virus with reduced glycosylation (16 to 32 HAU versus 2 HAU, respectively) (Fig. 4c). Therefore, HA with reduced glycan occupancy has less agglutinating ability per μg than does HA with normal glycosylation.

The enzymatic activities of glycome-modified and normal NA from culture supernatant A(H3N2)2013 IAV, normalized to viral mRNA, were compared in fluorometric and enzyme-linked lectin assays with 2′-(4-Methylumbelliferyl)-α-D-N-acetylneuraminic acid (MUNANA) or fetuin as a substrate, respectively. In contrast to HA receptor binding, NA enzymatic activities were similar between the two viruses with either substrate (Fig. 4d).

### Glycome-modified H3N2 IAV is less well recognized by the innate immunity respiratory tract collectin surfactant protein D.

Influenza virus HA and NA are important targets for virus neutralization by the respiratory tract innate immune collectins, particularly surfactant protein D (SP-D) (53). The carbohydrate recognition domain (CRD) of SP-D binds to high-mannose oligosaccharides in the vicinity of the receptor binding site of the HA globular head, especially to glycans attached to amino acids N165 and N246 (54). This binding by SP-D leads to neutralization and faster clearance of influenza virus (53, 55). Occupancy of the 3 N-glycosylation sites (N122, N165, and N246) with high mannose content on the HA globular head was substantially reduced in A(H3N2)2013 IAV from NGI-1-treated cells (Table 1).

SP-D is present in the respiratory tract in various oligomeric forms (56). Thus, we tested the ability of the full-length recombinant human (rh) SP-D monomer and dodecamer and two lower-affinity truncated versions of trimer (containing the neck and CRDs without or with binding-enhancing substitutions D325A/R343V) to inhibit hemagglutination (binding) of glycome-modified (with 10.0 μM NGI-1) or unmodified virus.

In hemagglutination inhibition (HI) tests, we found that unmodified A(H3N2) IAV was inhibited by much smaller amounts of monomeric, dodecameric, and trimeric/D325A/R343V rhSP-D compared to those of glycome-modified virus (from 1.25 μg/mL to >10 μg/mL for turkey RBC; from 0.63 to 5 μg/mL to 2.5 to 10 μg/mL for guinea pig RBC, respectively) (Table 2). As expected, none of the virus interactions with red blood cells were inhibited by truncated trimeric rhSP-D without mutations.

**TABLE 2:** Modified glycosylation of A(H3N2) IAV reduces HA binding by rhSP-D variants^*a*^

rhSP-D	Hemagglutinin-inhibiting concn (μg/mL) for:			
	Turkey red blood cells		Guinea pig red blood cells	
	NGI-1-treated virus	Untreated virus	NGI-1-treated virus	Untreated virus
Monomer	>10	1.25	5 (partial inhibition)	1.25
Dodecamer	>10	1.25	2.5	0.63
Truncated/ D325A/R343V	>10	1.25	10	5.0
Truncated	>10	10 (partial inhibition)	>10	>10

a Binding of the rhSP-D variants to glycome-modified HA from A(H3N2) IAV grown with or without 10.0 μM NGI-1 was tested in two independent HI tests with different lots of red blood cells. rhSP-D binding to virus HA was determined by measuring the concentration required to inhibit 4 HAU of NGI-1-treated or untreated viruses. Data from one representative experiment run in triplicate are shown.

Consistent with the reduced rhSP-D binding to HA, none of the tested rhSP-D doses was able to neutralize glycome-modified A(H3N2) IAV (Table 3). In contrast, virus with normal glycosylation (grown without NGI-1) showed a significant reduction in EID_50_/mL titers in the presence of 10 μg/mL of rhSP-D compared to either glycome-modified virus or unmodified virus without addition of rhSP-D.

**TABLE 3:** Modified glycosylation of A(H3N2) IAV reduces virus neutralization by rhSP-D variants^*a*^

rhSP-D (μg/mL)	Virus neutralization (EID_50_/mL) for:	
	NGI-1-treated virus	Untreated virus
10	10^4.2±0.2^	10^1.9±1.0^**
1.25	10^4.0±0.0^	10^3.8±0.4^
0.0	10^4.3±0.0^	10^4.0±0.0^

a Neutralization of glycome-modified A(H3N2) IAV (grown with 10.0 μM NGI-1) by rhSP-D was tested in two independent experiments run in duplicate. Cumulative data for rhSP-D monomer and dodecamer are shown and represent the average ± SD of the mean. An asterisk indicates significant differences in viral titers between NGI-1-treated (with modified glycome) and untreated (with normal glycome) virus incubated with rhSP-D or between virus with normal glycome incubated with or without rhSP-D by two-way ANOVA. **p < 0.005.

Therefore, A(H3N2) IAV with reduced glycan occupancy required higher rhSP-D concentrations for HA inhibition and virus neutralization than virus with normal glycan occupancy.

### Glycome modifications make H3N2 IAV less immunogenic.

Both HA- and NA-specific antibodies provide protection against influenza virus (57, 58). Each of the 5 antigenic sites of A(H3N2)2013 IAV HA was affected by NGI-1 treatment, since sites with reduced N-glycosylation were present in each (N45, C; N63, E; N122, A; N165, B; and N246, D) (Fig. 1b) (59, 60). In NA, three sites with reduced occupancy (N146, N200, and N367) were within the antigenic regions associated with residues 150/151, 152/198/199, and 342, respectively (50, 61).

We immunized naive mice with two intramuscular injections of concentrated purified A(H3N2)2013 IAV grown in the presence or absence of NGI-1 (Table 1), each containing 10 μg of HA. Serological responses were compared by enzyme-linked immunosorbent assay (ELISA) and by HI and neutralization assays. Antibody titers were measured against HA and NA antigens from the homologous virus A/Switzerland/9715293/2013(H3N2), as well as from heterologous H3N2 IAV isolated between 1968 and 2012 with various amounts of antigenic drift compared to the homologous strain.

**FIG 5:**
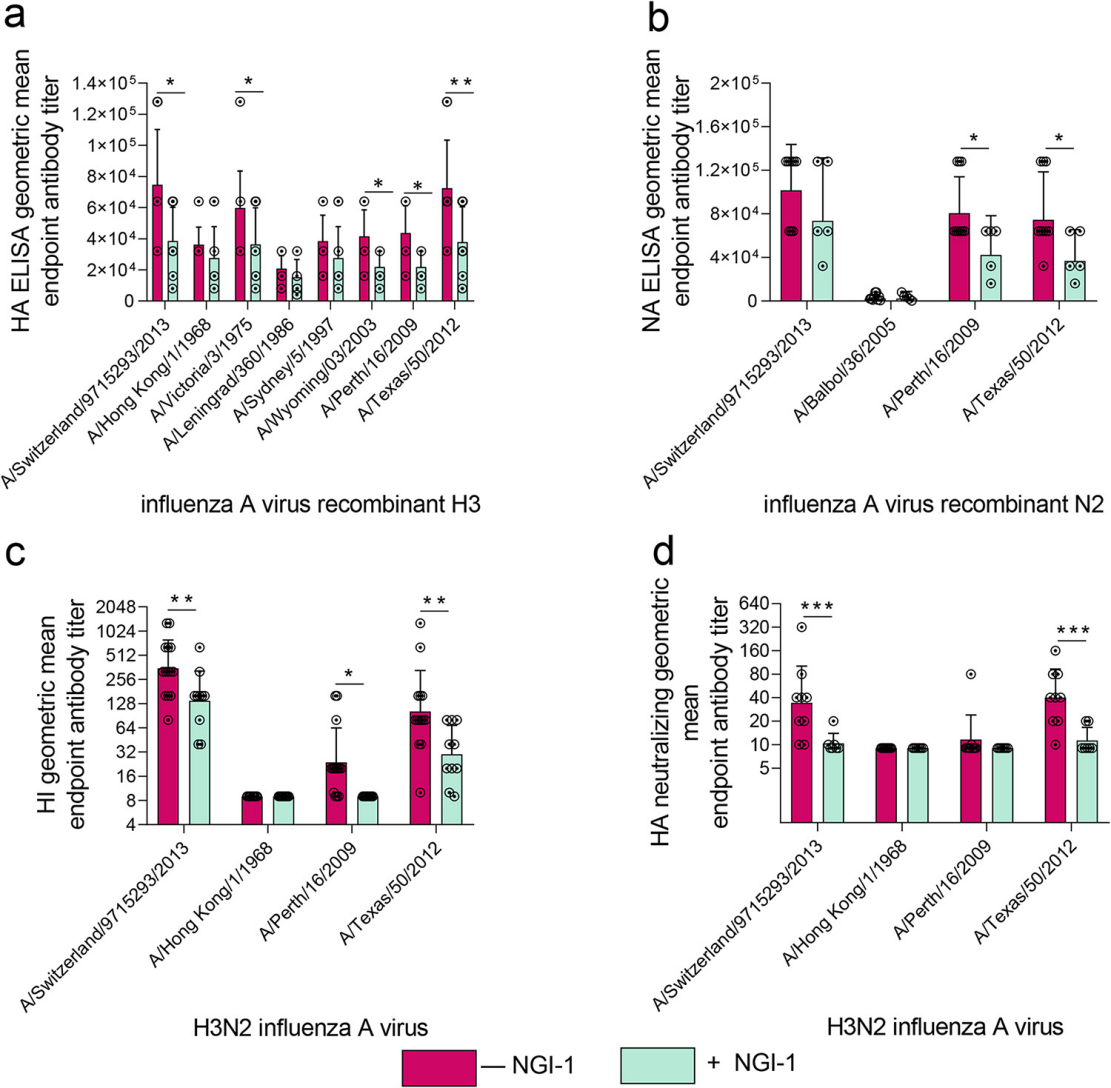
Glycome modifications reduce the immunogenicity of H3N2 IAV in mice. (a to d) Mice were immunized with 10.0 μg of HA from A(H3N2)2013 IAV with reduced or unmodified glycosylation of its HA and NA. (a and c) Pooled antibody titers from days 35 and 43 (*n* = 9 to 15) and (b) antibody titers for day 35 (*n* = 6 to 9) are shown. (a) HA and (b) NA total homologous and cross-reactive antibody titers were measured by ELISA using as antigen recombinant HAs and NAs from the indicated A(H3N2) IAV strain. (c and d) Homologous and cross-reactive HA antibody titers were determined by (c) HI and (d) neutralization assays with a panel of A(H3N2) IAV. Data represent the average (± SD) of two independent assays run in duplicate. Significant differences between groups: *, *P* < 0.05; **, *P* < 0.005; ***, *P* < 0.0005.

In general, anti-HA ELISA, HI, and HA neutralization titers were lower in mice immunized with virus with reduced N-glycosylation than in the mice immunized with the fully glycosylated virus (Fig. 5). Anti-HA ELISA titers, which measure all antibodies that bind to the protein, were less than half of those against homologous protein from A/Switzerland/9715293/2013(H3N2) (Fig. 5a). Cross-reactive antibody responses against heterologous H3N2 strains were also generally reduced in these mice. ELISA titers in serum raised against A(H3N2)2013 IAV with reduced glycosylation were decreased against HA proteins from the antigenically distinct strains A/Victoria/3/1975, A/Wyoming/03/2003, A/Perth/16/2009, and A/Texas/50/2012. Although ELISA titers against homologous NA were not significantly lower, cross-reactive antibodies against the NA proteins from A/Perth/16/2009 and A/Texas/50/2012 were reduced (Fig. 5b). HI and HA neutralization titers, which are correlated with protection against influenza disease, were also lower against HA of homologous virus in mice immunized with virus with reduced glycosylation (Fig. 5c and d, respectively). Cross-reactive HI antibodies were reduced by up to 4-fold against A/Perth/16/2009 and A/Texas/50/2012 H3N2 IAV (Fig. 5c), and cross-reactive HA neutralizing antibodies were reduced by up to 8-fold against A/Texas/50/2012 H3N2 IAV (Fig. 5d) in this group. Thus, HA and NA with reduced glycan occupancy generated lower antibody responses in mice than those with normal glycosylation.

Overall, our data suggest that aberrant cellular glycosylation can lead to sequence-neutral changes in the influenza virus glycome and that these glycome-modified viruses may be less well recognized by the host innate and adaptive immune system.

## DISCUSSION

People with metabolic disorders such as cancer, diabetes, obesity, or autoimmune diseases are more susceptible to severe influenza infections. Host factors such as reduced immune function have been implicated in this, since the viruses isolated from infections in these people do not have consistent sequence variations. Here, however, we show that altered host glycosylation, which is common in metabolic disorders, can cause physical and functional modifications in the infecting viruses, leading to production of progeny viruses that are relatively resistant to innate and adaptive immunity and which are therefore potentially more pathogenic.

We induced changes in host glycosylation that do not lead to cell death and which allow viral replication by treating cells with the small molecule OST inhibitor NGI-1 (36–38). This treatment led to the production of influenza viruses with lower N-glycosylation site occupancy and was associated with markedly reduced titers of multiple IAV and IBV strains (Fig. 2). Infectious titers of IBV viruses were reduced more than those of IAV with NGI-1 treatment. The hemagglutinin of IBV has been shown to be less structurally tolerant of mutations than that of IAV (62). It is likely that altered glycan composition similarly is more likely to affect the structure of IBV hemagglutinin. It is also possible that the structure of IBV HA makes it more dependent on the activity of OST for N-linked glycan addition to the two N-glycosylation (NLG) sites responsible for proper protein folding within the stem region (sites 22 and 38) than is IAV.

Levels of extracellular viral genomes of all tested viruses [except A(H3N2)2013 IAV] were reduced to a lesser extent than infectious virus (Fig. 3a and b), suggesting that NGI-1 treatment may lead to functionally defective virions, although A(H3N2)2013 virions were morphologically unaffected (Fig. 3d and e). Some virus subtypes [A(H1N1)pdm09 and IBV, but not A(H3N2)2013] also showed significantly lower levels of intracellular genomic RNA (Fig. 3c). This may be due to reduced virus infectivity leading to lower reinfection of cells or may be a direct effect on replication by NGI-1 as with Lassa virus (41). Together, these data suggest that entry, replication, and release of influenza virus may be affected by altered host NLG.

Genome sequencing did not reveal any mutations in the genome of glycome-modified A(H3N2)2013 IAV HA and NA. Mass spectrometry analysis showed reduced N-glycosylation site occupancy on HA and NA of this virus, especially in cases where sites are closely spaced, such as HA sites N38, N45, and N63, with the composition and subclass of glycoforms being unchanged (Table 1). These observations are consistent with previous findings that NGI-1 affects transfer of nascent glycans and not processing activities at later steps. STT3B, the subunit mostly inhibited by NGI-1, is involved in glycosylation of sites skipped by STT3A, which include closely spaced sequons (63). Influenza viruses, like Lassa virus, therefore seem to require STT3B for correct glycosylation (41).

The ability of HA to bind to its sialic acid receptors was reduced (Fig. 4c), while catalytic activity of NA was not affected (Fig. 4d), suggesting that NA functionality may be more resistant to glycosylation changes than is that of HA. Imbalanced HA and NA activities may, in part, explain the reduced infectious titers of the glycome-modified virus (Fig. 2 and 3), because a precisely tuned functional HA/NA equilibrium is required for efficient virus replication (64). Despite reduced receptor binding, A(H3N2)2013 IAV with modified glycosylation was able to infect new cells as well as was virus with normal glycosylation, consistent with previous observations that even low levels of HA binding can support efficient H3N2 IAV infection (65).

The surface proteins of influenza viruses, particularly the HA, have tended to gain N-glycosylation sites as they have adapted to human populations. Antibodies typically neutralize influenza infection by sterically blocking access of sialic acids to the HA and NA (23, 59, 66). By blocking antibody access to antigenic sites, glycans can protect viruses against immunity generated against previous strains (22, 23, 67). Reducing glycan site occupancy might be expected to increase virus immunogenicity by exposing antigenic sites to previously formed antibodies. However, in naive mice, we observed lower antibody titers to both HA and NA of glycome-modified viruses, for both homologous and heterologous viruses (Fig. 5).

Unlike mice, adult humans are rarely naive to influenza, having been exposed to multiple strains throughout their lives. The human serological response to influenza is therefore strongly affected by cross-reactivity with memory B cells induced by prior infections. Viruses grown in an imbalanced glycosylation environment therefore behave like antigenic drift variants even to genetically homologous strains, and as an even further drift variant to heterologous strains. Thus, reduced antibody responses to influenza infection in people with metabolic imbalances might be a combined result of viral antigenic and glycomic drift.

Although adding glycans may confer protection against immunity to previous virus strains, it probably also increases viral susceptibility to innate immunity as a result of neutralization by the respiratory tract immune collectins, such as SP-D (68). Consistent with this, A(H3N2)2013 IAV with reduced N-glycosylation site occupancy showed reduced inhibition and neutralization by rhSP-D (Tables 2 and 3). This may be one mechanism underlying the increased influenza viral load and viral shedding seen in people with metabolic disorders (69). Cytokine innate immune responses are not expected to be significantly different, since aberrant viral glycosylation should not directly influence recognition by innate pattern receptors for IAV, which are dominated by the nucleic acid receptor RIG-I, because the nucleic acid sequences in glycome-modified and unmodified A(H3N2)2013 IAV are identical.

Interestingly, people with congenital disorders of N-glycosylation may have decreased susceptibility to viral infections, including influenza virus infection. For example, siblings with a severe type IIb congenital disorder in the processing of N-glycans (resulting in multiple neurologic complications and severe hypogammaglobulinemia) showed only limited infection with influenza virus as a result of markedly impaired viral replication and cellular entry (70). The moderate changes in N-glycosylation of influenza virus (IV) induced by NGI-1, which may better model those present in metabolic disorders associated with cancer, diabetes or obesity, still allowed significant viral replication, spread between cells, and efficient infection of cell cultures with normal N-glycosylation. Interestingly, in contrast to IV, treatment with NGI-1 resulted in marked reduction of herpes simplex virus 1, resulting in a dysfunctional virus with reduced infectivity (40).

Together, our results show that aberrant cellular glycosylation may modify the biological properties of influenza viruses, with no changes in the virus genomic sequence, and that these viruses may be more pathogenic because they are less well recognized by both the innate and memory immune responses. Other respiratory viruses with glycosylated surface proteins, such as severe acute respiratory syndrome coronavirus 2 (SARS-CoV-2), might be similarly affected in people with metabolic disorders altering glycosylation, which may partially explain the observed increased severity of COVID-19 in cancer, type 2 diabetes mellitus, and in the obese.

Detailed investigation of changes in innate and adaptive cellular and antibody immunity caused by viruses with modified glycosylation is required for a comprehensive understanding of the mechanisms underlying increased severity and compromised immune response to respiratory viral infections in people with metabolic glycosylation imbalances.

## MATERIALS AND METHODS

### Inhibitor.

NGI-1 (5-dimethylsulfamoyl-N-5-methyl-1,3-thiazol-2-yl-2-pyrrolidin-1-yl-benzamide) was stored in dimethyl sulfoxide (DMSO) at −20°C prior to further dilution in tissue culture media for the experiment.

### Recombinant human SP-D (rhSP-D).

The rhSP-D precursor (surfactant protein [SFTPD]) was purchased from Sigma-Aldrich Corp. (St. Louis, MO, USA). The expression and preparation of rhSP-D dodecamer and trimers have been previously described (71, 72). Truncated trimers are composed of the neck and CRDs with or without the D325A and R343V substitutions on CRD (indicated as “truncated/D325A/R343V” and “truncated,” respectively) (73); the D325A and R343V substitutions in the truncated version of the rhSP-D allow its binding to IAV. The D325A/R343V double mutant was generated using the cDNA for the previously characterized R343V mutant (72). Site-directed mutagenesis was performed using a QuikChange II XL site-directed mutagenesis kit (Stratagene, La Jolla, CA, USA). RosettaBlue competent cells were transformed with the wild-type dodecamer or trimer or mutant trimer construct in pET-30a(+) vector, and expressed proteins were isolated from inclusion bodies. After refolding and oligomerization, fusion proteins were purified by nickel-affinity chromatography and then isolated by gel filtration chromatography on an AKTA purifier system. Endotoxin levels were less than ∼3 pg/μg of protein, including preparations used for the *in vivo* experiments. Proteins were stored in single-use aliquots at −80°C. Analytical gel filtration confirmed the absence of aggregation of the purified proteins under these storage conditions.

### Cells.

The epithelial cell line MDCK (Madin-Darby canine kidney) was purchased from American Tissue Culture Collection (ATCC, Manassas, VA, USA) and grown in 1× minimum essential medium (MEM; Thermo Fisher Scientific, Waltham, MA, USA) with 10% fetal bovine serum (FBS). An air-interface culture of differentiated primary normal human bronchial epithelial (NHBE) cells was purchased from MatTek Corporation (Ashland, MA, USA) and used for experiments in MatTek NHBE serum-free medium immediately after receiving.

### Viruses and recombinant proteins.

The following viruses were used in this study: (i) wild-type influenza viruses B/Brisbane/60/2008 (Victoria lineage) and (ii) B/Phuket/3073/2013 (Yamagata lineage); (iii) a reassortant virus termed “A(H3N2)2013” in this study containing PB2, PA, NP, M, and NS genes from A/Puerto Rico/8/34(H1N1), PB1 gene from A/Texas/1/1977(H3N2), and the HA and NA from A/Switzerland/9715293/2013(H3N2) (NIB-88, NIBSC code 14/314, National Institute for Biological Standards and Control, Potters Bar, Hertfordshire, UK); (iv) a reverse-genetics virus termed “A(H3N2)1968” in this study, with internal genes from A/Puerto Rico/8/34(H1N1), expressing the HA and NA genes of A/Hong Kong/1/1968(H3N2) (provided by J. McCullers, University of Tennessee Health Sciences Center, Memphis, TN, USA); and (v) a reverse-genetics virus termed “A(H1N1)pdm2009” in this study, with internal genes from A/Puerto Rico/8/34(H1N1), expressing the HA and NA of A/California/07/2009(H1N1pdm09). Viruses were grown either in 10-day-old embryonated chicken eggs or in MDCK cells. Full genomes were sequenced prior to experiments to confirm the absence of mutations.

Recombinant H3 or N2 proteins from homologous A/Switzerland/9715293/2013 or heterologous historical A/Hong Kong/1/1968, A/Victoria/3/1975, A/Leningrad/360/1986, A/Sydney/5/1997, A/Wyoming/03/2003, A/Perth/16/2009, and A/Texas/50/2012 H3N2 IAV were obtained either from the International Reagent Resource (IRR; Influenza Division, WHO Collaborating Center for Surveillance, Epidemiology, and Control of Influenza, Centers for Disease Control and Prevention, Atlanta, GA, USA; https://www.internationalreagentresource.org) or from J. Stevens, Centers for Disease Control and Prevention.

### Cell viability assay.

Assays determining NGI-1 cytotoxicity were performed with compound doses ranging from 0.1 μM to 30.0 μM. NGI-1 was added to the apical (MDCK) or basolateral (NHBE) cell surfaces for 24, 48, or 72 h. Then, cells were suspended and mixed with 0.4% trypan blue solution, and the dead (stained) cells and living (unstained) cells were counted using a Countess automated cell counter (Thermo Fisher Scientific). The concentration of the compound was considered nontoxic if the percentage of viable cells in NGI-1-treated cultures did not differ from that of untreated cultures.

### Virus infection of cultured cells.

MDCK or NHBE cells were infected with IAV and IBV at an MOI ranging from 0.01 to 1.0 to determine the effect of 10.0 μM NGI-1 on virus growth. NGI-1 was added to cell culture either 1 h before or 2 to 24 h postinfection (p.i.). MDCK cells in 24-well plates were infected with influenza virus for 1 h at 37°C. After virus adsorption, cells were washed 3 times with phosphate-buffered saline (PBS), pH 7.2, and then incubated at 37°C for the duration of the experiment in infection medium (MEM supplemented with bovine serum albumin) containing 1 μg/mL acetylated trypsin. In the case of NHBE cells, apical secretions were removed prior to infection, and cells were supplied with fresh basolateral MatTek serum-free medium. Virus was added to the apical surface of cells in a volume of 300 μL for 1 h at 37°C. Following incubation, monolayers were washed with PBS to remove nonadherent virus. The apical surface of cells was washed with 300 μL of medium for 30 min at 37°C at the indicated times to collect virus.

The amount of virus in the apical washes from NHBE cells or tissue culture supernatants from MDCK cells was determined by TCID_50_ in MDCK cells or by EID_50_ assays as described elsewhere (74).

### Reverse transcription quantitative PCR (RT-qPCR).

RT-qPCR was performed 24 h p.i. to quantify A(H3N2)2013 IAV genomes in the lysates or apical washes from NHBE cells untreated or 1-h pretreated with 10.0 μM NGI-1. Total RNA from cells was extracted from cells using an RNeasy Plus minikit (Qiagen, Germantown, MD, USA) according to the manufacturer’s instructions. Briefly, cells from a single well were pelleted and resuspended in 600 μL RLT lysis buffer. The sample was vortexed and run through a QIAshredder column. Total RNA from the apical wash was extracted using a QIAamp Viral RNA minikit (Qiagen) according to the manufacturer’s instructions. RNA purity was assessed using a NanoDrop 1000 spectrophotometer (Thermo Fisher Scientific) and an *A*_260_/*A*_280_ ratio of absorbance. Reverse transcription and quantitative PCR were performed with a SuperScript III Platinum one-step RT-qPCR kit (Thermo Fisher Scientific), and primers and probes were from the CDC influenza virus real-time RT-PCR influenza A/B typing panel for research use only (catalog no. FluRUO-01), FR-198, obtained through the IRR. DNA plasmid controls with known DNA concentration were included on each plate, and the same mastermix preparation was used for all RT-qPCR plates. Plasmid controls were used to create a normalization curve which was used to determine the RNA concentration in the samples.

### Negative stain electron microscopy (EM).

EM was performed to quantify the number of total and morphologically typical virions in apical washes from NHBE or culture supernatants from MDCK cells either pretreated for 1-h with 10.0 μM NGI-1 or left untreated and then infected with A(H3N2)2013 IAV at an MOI of 2.0. After 24 h, virus-containing washes or supernatants from three independent wells were collected and fixed in 2.5% paraformaldehyde. Virus from each well was adsorbed onto a Formvar/carbon-coated grid, stained with 5% ammonium molybdate/0.1% trehalose, and examined with a Tecnai BioTWIN electron microscope (Thermo Fisher/FEI). The number of total and morphologically typical virions was counted for at least six grid openings (58.0 μ by 58.0 μ) per well, with a total of at least 18 grid openings per treatment being examined.

### Gel electrophoresis and immunoblot analyses.

For gel electrophoresis, MDCK cells in 6-well plates were pretreated with 0.1 to 10.0 μM NGI-1 for 1 h, or mock treated, and infected with virus at an MOI of 1.0. After incubation at 37°C for 24 h, the cells were harvested and lysed in Laemmli buffer (Sigma-Aldrich). Extracts were centrifuged (15,700 × *g*, 15 min), and proteins were denatured by heating at 70°C for 10 min in 1× sodium dodecyl sulfate-polyacrylamide gel electrophoresis (SDS-PAGE) NuPAGE LDS sample buffer (Invitrogen, Carlsbad, CA, USA). The migration patterns of NGI-1-treated versus untreated HA/NA protein aggregates were compared under nonreducing conditions in NuPAGE 10% Bis-Tris protein gel after staining with SimplyBlue SafeStain (Invitrogen). The imaging was accomplished using an Perfection 2400 photo scanner (Epson, Long Beach, CA, US), and the densities of the HA/NA bands were analyzed with Gel Eval (v1.37; FrogDance Software, United Kingdom).

For immunoblot analysis, A(H3N2)2013 IAV was grown on MDCK cells in the presence or absence of 5.0 μM NGI-1 and was concentrated and purified through a gradient of 30% to 50% sucrose in PBS. Virus proteins were denatured in the presence of NuPAGE LDS reducing agent (Invitrogen) and separated by SDS-PAGE and transferred to nitrocellulose membrane which was then probed with an anti-H3 mouse monoclonal antibody targeting the HA1 subunit of H3N2 A/Switzerland/975293/2013 IAV HA (eEnzyme LLC, Gaithersburg, MD, USA) and a peroxidase-labeled secondary goat anti-mouse antibody (Sigma-Aldrich).

### Glycomics.

Mass spectrometry (MS) analysis was performed to assess glycosylation changes on concentrated purified A(H3N2)2013 IAV grown in the presence of 5.0 μM NGI-1. Prior to analysis, NGI-1-treated and untreated viruses were inactivated with β-propiolactone (BPL; Sigma-Aldrich) and then washed three times with PBS to remove the BPL (75). Three analytical replicates of reverse-phase nanoscale liquid chromatography coupled with MS analysis of glycopeptides and peptides were performed as described previously (76). As an inherent property of the A(H3N2)2013 IAV HA and NA, in some cases, the tryptic peptides analyzed in our experiments for both glycoproteins tended to contain multiple N-glycosylation sites in some tryptic glycopeptides. For instance, HA N-glycosylation sites N8 and N22 reside on one glycopeptide, as do N38, N45 and N63, as well as N122 and N133 and NA sites N61 and N70. Where the glycosites were not discernible, the data are presented such that the detected glycan compositions can be at either site.

Briefly, the chromatography system consisted of a C_18_ column (bridged-ethyl hybrid [BEH] nanocolumn 100 μm by 100 mm inside diameter [i.d.], 1.7-μm particle; Waters Corp., Milford, MA, USA) installed into a Waters nanoAcquity ultraperformance liquid chromatography (UPLC) system with automatic sample loading and flow control. Load buffer was 3% acetonitile and 97% water. Peptides were eluted via a 60-min gradient from 3 to 50% acetonitile with a flow of 0.4 μL/min. All chromatography solutions included 0.1% formic acid. The eluent flowed to an uncoated 20-μm i.d. PicoTip emitter (New Objective, Inc., Woburn, MA, USA) for electromotive emission to the mass spectrometer, a Waters SYNAPT G2 high-definition mass spectrometer (HDMS) system. The applied source voltage was 3,000 V. Data were collected in positive polarity mode using data-independent MS^E^ acquisition, which consists of a starting 4-V scan followed by a scan ramping from 20 to 50 V in 0.9 s (77). To calibrate internally, every 30 s, 400 fmol/μL glufibrinopeptide B with 1 pmol/μL leucine enkephalin in 25% acetonitrile, 0.1% formic acid, and 74.9% water were injected through the lockmass channel at a flow rate of 500 nL/min. Initial calibration of the mass spectrometer was performed in MS2 mode using glufibrinopeptide B and tuned for a minimum resolution of 20,000 resolving power using full width at half maximum.

For site occupancy analysis, LC/MS^E^ data were collected on trypsinized peptides deglycosylated with PNGase F. Asn deglycosylated by peptide-N-glycosidase F (PNGase F) was converted to Asp with a mass gain of 0.984 Da due to the replacement of –NH2 with –OH. The percent occupancy for each site is calculated by comparing the intensity of peptides with Asn to those with Asp. Percent occupancy was calculated by comparing the intensities of the deglycosylated (DG) and nonglycosylated (NG) peptides using the equation DG/(DG+NG) · 100. The program NetNGlyc v1.0 (with an overall accuracy of 76%) was used to estimate the probability of glycosylation events at each glycosylastion site for both HA and NA (78). A NetNGlyc potential score of 0.5 or higher indicates a high potential for occupancy. The Peptide Synthesis and Proteotypic Peptide Analyzing Tool (Thermo Fisher; https://www.thermofisher.com/us/en/home/life-science/protein-biology/peptides-proteins/custom-peptide-synthesis-services/peptide-analyzing-tool.html) was used to determine the hydrophobicity.

### Immunization of mice.

Immunization was completed to evaluate antibody responses generated toward glycome-modified A(H3N2)2013 IAV. Experiments using 8-week-old female BALB/c mice (Jackson Laboratories, Bar Harbor, ME, USA) were performed in a biosafety level 2 facility in the Animal Resources Center at the CDC. Animals were anesthetized with 2.5% inhaled isoflurane (Baxter Healthcare Corporation, Deerfield, IL, USA) prior to all interventions. Studies were conducted under CDC IACUC-approved protocols and in accordance with the relevant guidelines and regulations.

Ten mice per group were immunized intramuscularly twice at 21-day intervals with 10.0 μg of HA from concentrated purified BPL-inactivated A(H3N2)2013 IAV grown in the presence or absence of 5.0 μM NGI-1 in sterile PBS, or with sterile PBS as a control. Total protein and HA in the preparations were measured using the Bio-Rad protein assay (Bio-Rad, Hercules, CA, USA) and reducing SDS-PAGE (respectively). Blood samples were collected on days 0 (before immunization), 35, and 43.

### Mouse serum preparation.

Sera were treated with receptor-destroying enzyme (RDE; Denka Seiken Co. Ltd., Tokyo, Japan) for 18 h at 37°C (resulting in an initial serum dilution of 1:10), and the enzyme was then heat-inactivated at 55°C for 30 min. RDE-treated sera were adsorbed with packed turkey red blood cells at 4°C for 30 min to remove nonspecific agglutinins and then were stored at −80°C. Sera were thawed immediately before serological assays.

### Hemagglutination and hemagglutination inhibition (HI) assays.

Hemagglutination assays were run to determine the receptor binding abilities of concentrated purified A(H3N2)2013 IAV grown in MDCK cells without or with 5.0 μM NGI-1. Virus was diluted in PBS to a total protein concentration of 0.001 μg/μL, and hemagglutination titers, expressed in HAU, were determined with 0.5% chicken or turkey or 0.75% guinea pig red blood cells after incubation for 1 h at 4°C as described elsewhere (74).

HI assays were completed to measure the binding of the rhSP-D to HA and the levels of HA-specific neutralizing antibodies in the immune mouse sera as described elsewhere (74). rhSP-D and sera were tested against 4 HAU per 25 μl of virus.

In assays determining rhSP-D binding, 10 μg/mL of precursor, dodecamer, and two truncated versions of the rhSP-D protein with or without the double D325A+R343V mutation were serially diluted in Dulbecco’s (D-)phosphate-buffered saline containing Ca^2+^ and Mg^2+^ (Quality Biological, Inc., Gaithersburg, MD, USA) in 96-well plates. A(H3N2)2013 IAV grown in MDCK cells with or without 10.0 μM NGI-1 was incubated with serial dilutions of rhSP-D for 30 min at room temperature. In parallel, virus was incubated with PBS. Suspension of turkey or guinea pig red blood cells was added to the mixture. The plate was incubated for 45 min, and the rhSP-D concentration required for HI was recorded. If no HI appeared at the highest protein concentration used, then the value was expressed as greater than the maximal rhSP-D concentration tested.

In assays measuring titers of HA neutralizing antibodies, sera from immunized mice were serially 2-fold diluted and incubated with 4 HA of tested virus for 30 min at room temperature. Agglutination was detected with turkey red blood cells. HI titers were defined as the reciprocal of the last dilution of serum that completely inhibited hemagglutination. In the case of neutralization assays, sera from immunized mice were serially 2-fold diluted and incubated with 100 TCID_50_/50 μl of tested virus for 1 h at 37°C. Then, MDCK cells in 96-well plates were inoculated with the virus antisera mixture and incubated for 48 h at 37°C. The presence of virus was determined by HA tests with 0.5% turkey RBC. The minimum detectable limit of either assay was a titer of 10. Samples with titers of <10 were assigned a value of 9 for calculating geometric mean titers (GMT).

### Virus neutralization assays.

Assays were performed to determine the ability of the rhSP-D to neutralize A(H3N2)2013 IAV grown in MDCK cells without or with 10.0 μM NGI-1. rhSP-D precursor or dodecamer was diluted to 10 μg/mL and 1.25 μg/mL in D-phosphate-buffered saline containing Ca^2+^ and Mg^2+^ (Quality Biological). NGI-1-treated or untreated A(H3N2)2013 IAV (10^4.0^ EID_50_/mL) was incubated with rhSP-D dilutions for 1 h at room temperature. In parallel, virus was incubated with PBS. The amount of virus in infected 10-day-old embryonated chicken eggs was determined 48 h p.i. by EID_50_ assays.

### Enzyme activity assays.

Activity of the NA protein from A(H3N2)2013 IAV grown in MDCK cells in the presence or absence of 10.0 μM NGI-1 was analyzed either by a fluorometric assay using 2′-(4-methylumbelliferyl)-α-d-*N-*acetylneuraminic acid (MUNANA; Sigma-Aldrich) as the substrate, or by an enzyme-linked lectin (ELLA) assay using fetuin (Sigma-Aldrich) as the substrate (79, 80). The fluorometric assay was performed using the NA-Fluor influenza neuraminidase assay kit (Applied Biosystems/Life Technologies, Carlsbad, CA, USA) in 96-well opaque black flat-bottom microplates (Corning, Inc., Corning, NY, USA). Briefly, 2-fold-diluted virus was mixed with 200 μM NA-Fluor substrate (MUNANA) and incubated at 37°C for 60 min. The reaction was terminated with NA-Fluor sTOP solution, and fluorescence was detected on the BioTek plate reader platform (models Synergy H1, Synergy Neo, and Cytation 3; BioTek Instruments, Inc., Winooski, VT, USA) using excitation and emission wavelengths of 365 and 460 nm, respectively. The enzyme kinetic data were fit to the Michaelis-Menten equation by using nonlinear regression to establish the Michaelis constant and the maximum velocity of substrate conversion.

For the ELLA assay, 2-fold-diluted virus was added to fetuin-coated flat-bottom 96-well Nunc MaxiSorp plates (BioLegend, San Diego, CA, USA) for 18 h of incubation at 37°C. Following incubation, horseradish peroxidase-labeled peanut agglutinin (lectin) was added to the reaction mixture and incubated for 2 h, followed by 3,3′,5,5′-tetramethylbenzidine (TMB) substrate to reveal enzymatic cleavage of fetuin by viral NA. The signal was read using a 450- nm emission wavelength. In both assays, the relative NA activity is activity per viral mRNA copy.

### Enzyme-linked immunosorbent assay (ELISA).

ELISAs were completed to measure HA- and NA-specific total antibody titers. ELISA plates were coated overnight at 4°C with 2.0 μg/mL of recombinant H3 or N2 proteins and blocked for 2 h with PBS containing 0.1% fetal bovine albumin (BSA; Sigma). Then, plates were washed three times with PBS containing 0.1% Tween 20 (PBS-T), and 50 μL of 2-fold serial dilutions of 1:1,000 serum samples were added to each well. After 2 h of incubation, plates were washed three times with PBS-T, and then 50 μL of peroxidase-conjugated anti-mouse immunoglobulin (Ig) G (Alpha Diagnostic, San Antonio, TX, USA) diluted in PBS containing 3% BSA was added to each well. After a 1-h incubation, plates were washed three times with PBS-T. O-phenylenediamine (OPD) solution and H_2_O_2_ were used as the substrate to develop the plates, and hydrochloric acid was used to stop the reaction. Plates were read at an optical density (OD) of 490 nm using the SpectraMax 190 microplate reader (Molecular Devices). GMT antibody titers are given as the reciprocal of the highest dilution which gave an OD_490_ value of >2 times the average of the background wells.

### Statistical analysis.

Analysis of variance (ANOVA) followed by Tukey’s multiple-comparison test was used to estimate and compare the viral titers in NGI-1-treated versus untreated tissue culture supernatant samples. NA activities and NLG site occupancy of and frequency of morphologically normal and abnormal NGI-1-treated versus untreated viruses were compared using Student’s unpaired *t* test. A linear mixed model with repeated measures was used to compare the immunogenicity of NGI-1-treated versus untreated HA. A *P* value of <0.05 was considered significant for all comparisons. Statistical analyses were performed using GraphPad Prism 8.0, Excel 2019, and SAS 9.4.

10.1128/mBio.02983-21.3 FIG S3 Graphical abstract. Download FIG S3, TIF file, 2.6 MB. Copyright © 2022 Alymova et al. 2022 Alymova et al. https://creativecommons.org/licenses/by/4.0/ This content is distributed under the terms of the Creative Commons Attribution 4.0 International license.
