# Supplementary figures and images for: Aberrant Cellular Glycosylation May Increase the Ability of Influenza Viruses to Escape Host Immune Responses through Modification of the Viral Glycome
Source: mBio. 2022 Mar 14;13(2):e02983-21. doi: 10.1128/mbio.02983-21 (PMC9040841; doi:10.1128/mbio.02983-21)

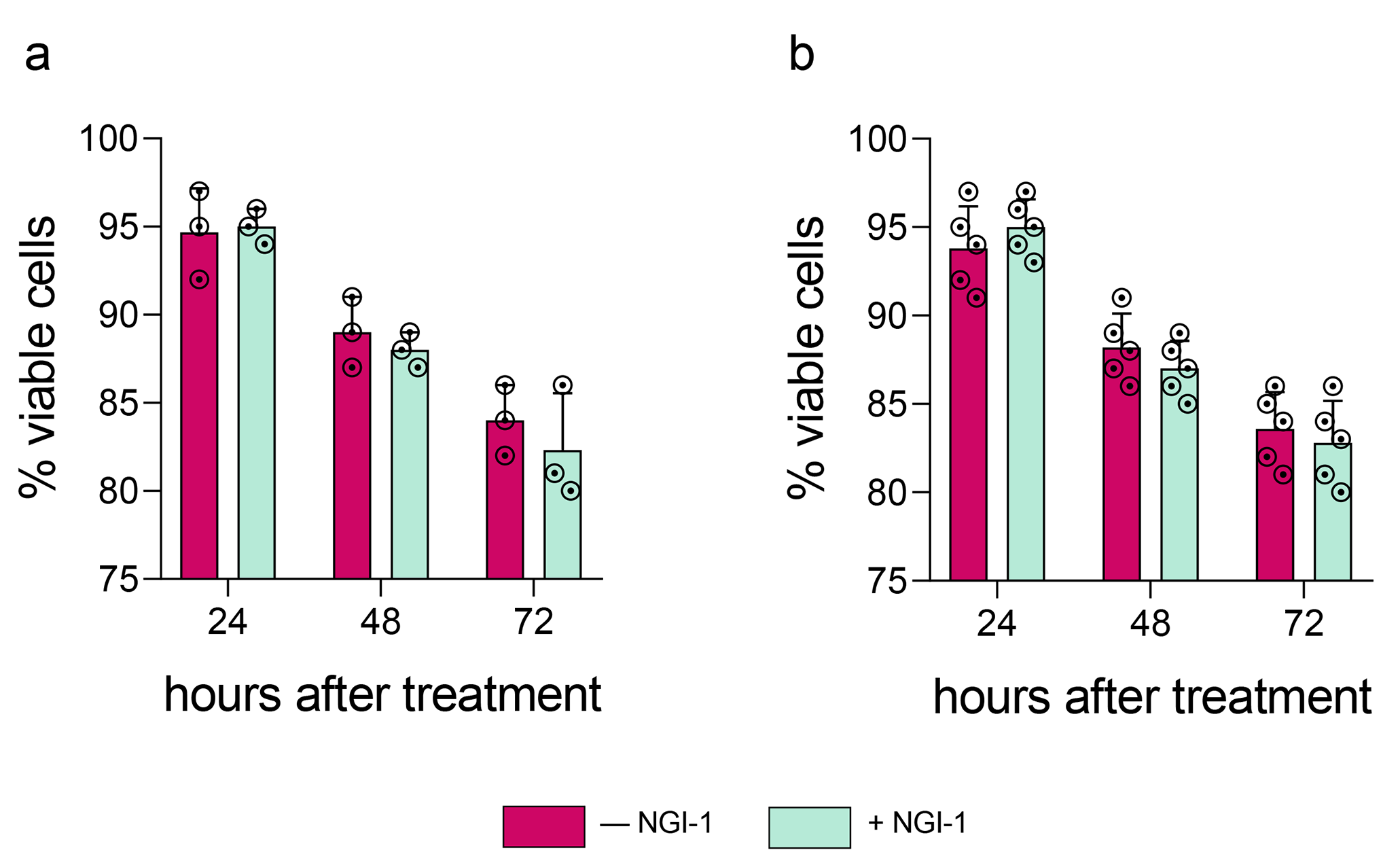

Supplement: FIG S1 [file mbio.02983-21-sf001.tif]

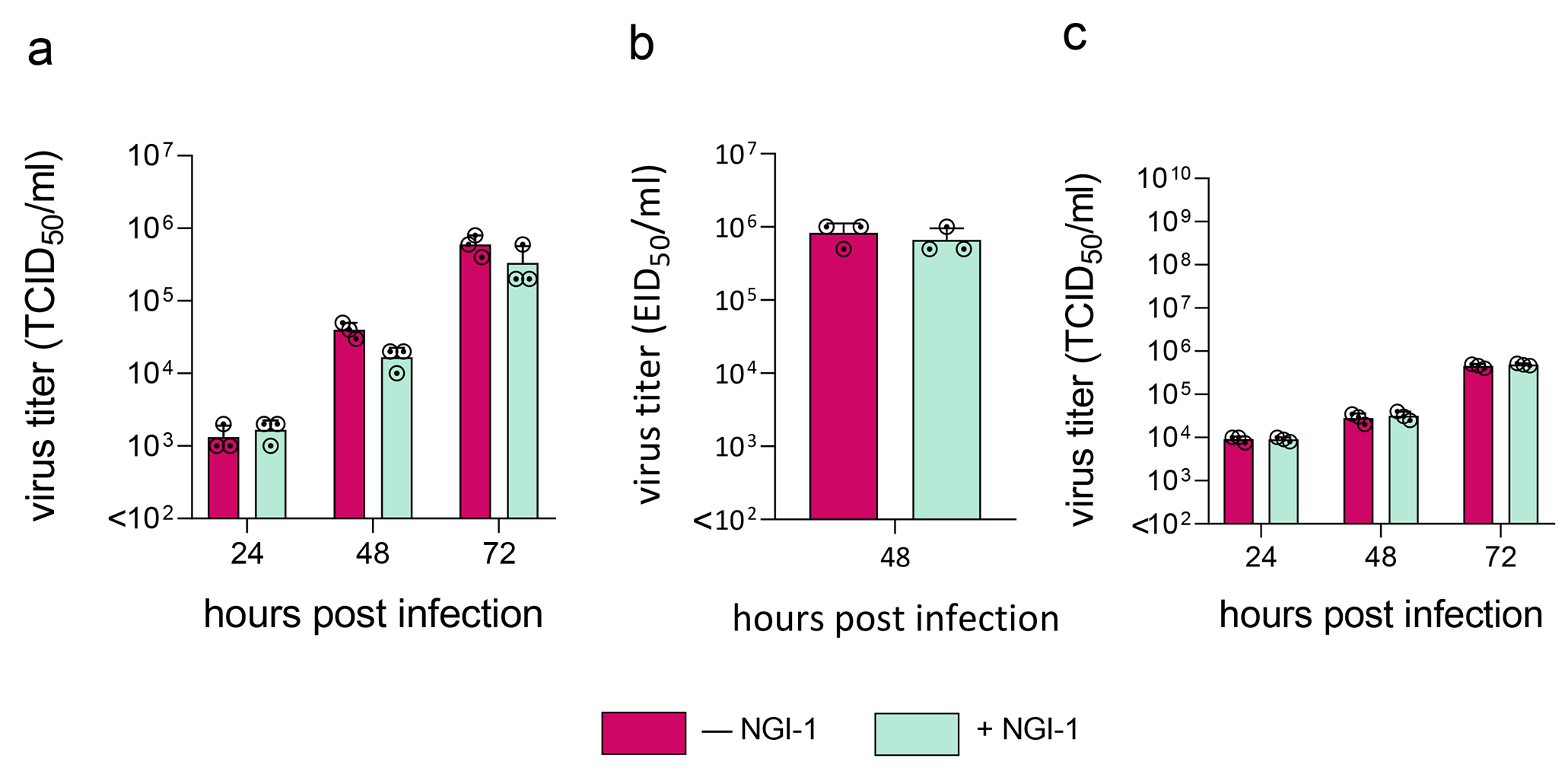

Supplement: FIG S2 [file mbio.02983-21-sf002.tif]

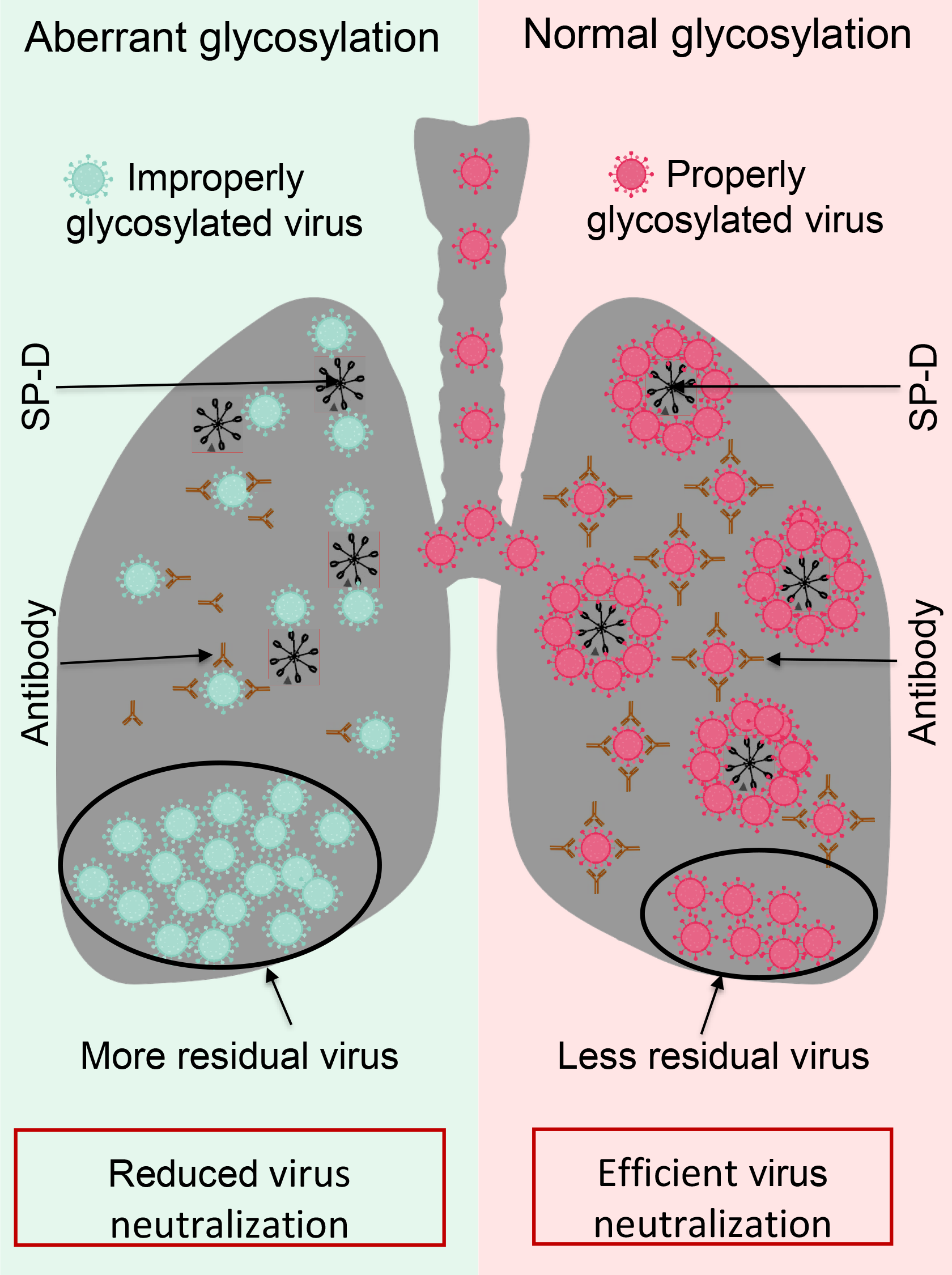

Supplement: FIG S3 [file mbio.02983-21-sf003.tif]
